# A Rare Chromosomal Abnormality in Chronic Lymphocytic Leukemia: t(13;13)

**DOI:** 10.4274/tjh.galenos.2019.2019.0134

**Published:** 2020-05-06

**Authors:** Akbar Safaei, Ahmad Monabati, Moeinadin Safavi

**Affiliations:** 1 Shiraz University of Medical Sciences Molecular Pathology and Cytogenetic Section, Medical Faculty, Department of Pathology, Shiraz, Iran; 2 Tehran University of Medical Sciences Medical Faculty, Department of Pathology, Tehran, Iran; 3 Shiraz University of Medical Sciences, Hematopathology Research Center, Shiraz, Iran

**Keywords:** Chronic lymphocytic leukemia, Cytogenetics, Chromosome 13

The patient was a 67-year-old man with peripheral blood lymphocytosis. The patient’s complete blood count revealed hemoglobin of 12.2 g/dL, white blood cell count of 22,000/µL, and platelet count of 124,000/µL. The differential count for white blood cells was as follows: neutrophils, 10%; lymphocytes, 86%; and monocytes, 4%. Absolute lymphocyte count was 18,920/µL. Flow cytometry of peripheral blood revealed 86% lymphocytes, which were positive for CD19, CD79b, CD20 (dim), CD5, CD23, and CD45, but they were negative for FMC7 and CD38. Blood culture with phorbol 12-myristate 13-acetate (TPA) and subsequent Giemsa banding revealed t(13;13)(q14;q32)[8]/46,XY[12] (Figure 1).

Structural aberrations of the long arm of chromosome 13,t/del(13q) account for 20% of all chromosomal abnormalities in chronic lymphocytic leukemia [1]. This rate is even higher when more precise methods like fluorescence in situ hybridization are used for deletion of band 13q14, reaching 50% of all cases. Although translocations of chromosome 13 could have different counterparts, t(13;13) has been reported very rarely. According to the Mitelman database, only six cases have been registered so far [2].

## Figures and Tables

**Figure 1:**
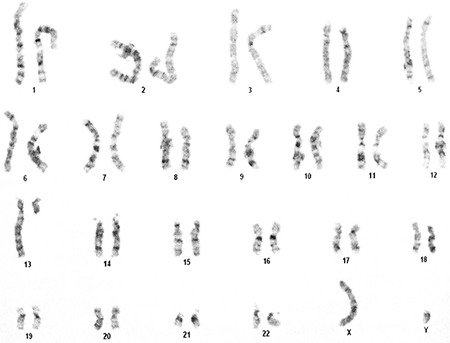
Peripheral blood culture with TPA revealed t(13;13) (q14;q32).
